# Natural Compounds from Herbs that can Potentially Execute as Autophagy Inducers for Cancer Therapy

**DOI:** 10.3390/ijms18071412

**Published:** 2017-07-01

**Authors:** Shian-Ren Lin, Yaw-Syan Fu, May-Jywan Tsai, Henrich Cheng, Ching-Feng Weng

**Affiliations:** 1 Department of Life Science and Institute of Biotechnology, National Dong Hwa University, 97401 Hualien, Taiwan; d9813003@gms.ndhu.edu.tw; 2 Department of Biomedical Science and Environmental Biology, Kaohsiung Medical University, 807 Kaohsiung city, Taiwan; m805004@kmu.edu.tw; 3 Neural Regeneration Laboratory, Department of Neurosurgery, Neurological Institute, Taipei Veterans General Hospital, 11221 Taipei, Taiwan; mjtsai2@vghtpe.gov.tw (M.-J.T.); hc_cheng@vghtpe.gov.tw (H.C.); 4 Center for Neural Regeneration, Neurological Institute, Taipei Veterans General Hospital, 11221 Taipei, Taiwan

**Keywords:** autophagy inducer, autophagy inhibitor, natural compound, cancer therapy

## Abstract

Accumulated evidence indicates that autophagy is a response of cancer cells to various anti-cancer therapies. Autophagy is designated as programmed cell death type II, and is characterized by the formation of autophagic vacuoles in the cytoplasm. Numerous herbs, including Chinese herbs, have been applied to cancer treatments as complementary and alternative medicines, supplements, or nutraceuticals to dampen the side or adverse effects of chemotherapy drugs. Moreover, the tumor suppressive actions of herbs and natural products induced autophagy that may lead to cell senescence, increase apoptosis-independent cell death or complement apoptotic processes. Hereby, the underlying mechanisms of natural autophagy inducers are cautiously reviewed in this article. Additionally, three natural compounds—curcumin, 16-hydroxycleroda-3,13-dien-15,16-olide, and prodigiosin—are presented as candidates for autophagy inducers that can trigger cell death in a supplement or alternative medicine for cancer therapy. Despite recent advancements in therapeutic drugs or agents of natural products in several cancers, it warrants further investigation in preclinical and clinical studies.

## 1. Introduction

Cancer is a group of diseases involving out-of-control of cell growth due to the accumulation of defects, or mutations, in their DNA and with an impendence to invade or spread to other parts of the body [1]. In 2015, about 90.5 million people were diagnosed with cancer [2]. About 14.1 million new cases occur each year (not including skin cancer other than melanoma) [3]. Consequently, it causes about 8.8 million (15.7%) human deaths [4]. Anti-cancer drugs including 5-fluorouracil (5-FU), cisplatin, etoposide, paclitaxel, and doxorubicin are commonly used to treat various cancers, such as cisplatin and doxorubicin in ovarian cancer, 5-FU in colon and gastric cancer, paclitaxel and doxorubicin in breast cancer, and etoposide in small-cell lung cancer. However, these chemotherapeutic agents have evident side effects such as nausea, vomiting, loss of appetite, decreased immunity, oral ulcers, and other adverse effects [5]. In general, the anti-cancer drugs, such as cisplatin and doxorubicin favor abnormal triggering via programmed cell death (PCD) such as apoptosis, necrosis, necroptosis, and autophagy in normal cells as well as abolishing inflammation of damaged cells. Remarkably, apoptosis and autophagy are traditionally considered the most prominent cell death or cell death-related mechanisms for anti-cancer drug discovery [6,7].

In contrast to apoptosis, autophagy is a homeostatic process, and is one of the earliest responses to pharmacologically active compounds lead to ultra-structural perturbations; changes in membrane composition, cytoskeleton integrity, alterations of the endoplasmic reticulum, mitochondria and nucleus all result in the formation of morphological alterations. This biological process of autophagy is designated as type II PCD, and has several features, such as removing damaged or excessive proteins, utilization by various types of cells to maintain cellular homeostasis, high conservation across different types of mammalian cells, and is a potentiated cancer type preceding cell death. Moreover, autophagy can relieve tumor cells from nutrient and oxidative stress during a rapid expansion of a cancer. Excessive and sustained autophagy may lead to cell death and tumor shrinkage. Interestingly, autophagy also occurs in cancer cells and exerts anti-survival or pro-survival effects depending on stimuli, nutrients, and context.

## 2. The Significance of Autophagy in Cancer

Autophagy is an important process in many functions such as maintaining protein quality, presenting antigens, responding to endoplasmic reticulum (ER) stress, and supplying energy [8]. These functions represent a stress in cells so that autophagy usually links to degenerative diseases like dementia and Parkinson’s disease [9]. There are two opposite mechanisms for autophagy in cancer development. Firstly, autophagy can clean up damaged organelles or protein accumulations and further activate programmed cell death when the cell is severely damaged. This anti-cancer mechanism provides another checkpoint to avoid tumorigenesis. On the other hand, autophagy also affords a proportional way of overcoming chemotherapeutic drugs and a low energy state. Autophagy could be activated by ER stress and some reactive oxygen species (ROS), inducing chemotherapeutic drugs like cisplatin and doxorubicin, where it can assist cancer stem cells in cleaning up ROS-damaged proteins [10]. Autophagy, a catabolic degradation process via lysosomes, plays an important role in tumorigenesis and cancer therapy.

### 2.1. Natural Autophagy Inhibitor from Herbs

Currently, many herbs, including Chinese herbs, have been applied as cancer treatments to complement and act as alternative medicines, supplements, or nutraceuticals to dampen the aforementioned problems. It has been shown in the literature that many anti-cancer natural compounds and extracts could initiate autophagy in tumor cells. As summarized in this paper, tumor suppressive actions of natural product-induced autophagy may lead to cell senescence, provoke apoptosis-independent cell death, and complement apoptotic cell death by robust or target-specific mechanisms. Notably, natural compounds are fundamental for pharmacological treatments, and more than 50% of all anti-cancer drugs are of natural origins, or at least derived from scaffolds present in nature. Emerging research shows that molecules of natural origins are useful for preventive and therapeutic purposes by targeting essential hallmarks and enabling described characteristics. Moreover, natural compounds can change the differentiation status of selected cell types. In the past decade, some autophagy-related inducers and inhibitors have been extensively investigated. Of note, some autophagy inhibitors including 3-methyladenine (3-MA), pepstatin A, bafilomycin A1, and betulinic acid have been well studied, and have been applied as suppressors to examine the modality of autophagy formation in autophagy research [11]. Curiously, other natural autophagy inhibitors have also been elucidated as: (1) Matrine, a natural compound extract used in traditional Chinese medicine, can modulate the maturation process of lysosomal proteases in gastric cancer cell line (SGC-7901) cells [12]; (2) Elaiophylin promotes autophagosome accumulation but blocks autophagic flux by attenuating lysosomal cathepsin activity, resulting in the accumulation of SQSTM1/p62 in various human ovarian cancer cell lines [13]; (3) Oblongifolin C (OC), a natural small molecule compound extracted from *Garcinia yunnanensis* Hu, is a potent autophagic flux inhibitor. Exposure to OC results in an increased number of autophagosomes and impaired degradation of SQSTM1/p62 [14]; (4) p53 siRNA and epigallocatechin gallate (EGCG) dual therapy leads to the activation of pro-apoptotic genes, the inhibition of pro-survival autophagy and cell network formation [15]; (5) Frondoside A, a triterpenoid saponin with a sugar-steroid structure, is derived from the orange-footed sea cucumber, *Cucumaria frondosa* and inhibits pro-survival autophagy, a known mechanism of drug resistance in the human urothelial carcinoma cell lines and showed the synergistic activity with cisplatin and gemcitabine [16]; and (6) Rhizochalinin (Rhiz) from the marine sponge *Rhizochalina incrustata*, a novel sphingolipid-like marine compound is characterized by a unique combination of anti-cancer properties via one scenario of pro-survival autophagy inhibition in the human prostate cancer cells [17]. Thereby, natural products have also been demonstrated as autophagy inducers.

### 2.2. Natural Autophagy Inducers from Herbs

Conversely, the autophagy cascade may play a regulatory or major role resulting in cell death, particularly when natural products are employed. Apparently, more recent studies have elicited the benefits and molecular mechanisms triggered by natural active components for anti-cancer activity, particularly in the induction of autophagy. Sirolimus, also known as rapamycin, is isolated from the bacterium *Streptomyces hygroscopicus*, and may be the most famous natural autophagy inducer in autophagy research [18]. The extract of *Emblica officinalis* (Amla) inhibits ovarian cancer (OC) cell growth in vitro and in vivo, possibly via inhibition of angiogenesis and activation of autophagy in OC [19]. The natural compound lipoic acid (LA) inhibits O (6)-methylguanine-DNA methyltransferase (MGMT) and induces autophagy and subsequently LA enhances the cytotoxic effects of temozolomide in HCT-116 cells [20]. The combination of gossypol and BRD4770 increased LC3-II levels and the autophagosome number in PANC-1 cells. The compound combination appears to act in a BNIP3 (B-cell lymphoma 2 19-kDa interacting protein)-dependent manner, suggesting that these compounds act together to induce autophagy-related cell death in pancreatic cancer cells [21]. The synthesized natural alkaloid berberine derivatives are able to induce autophagy for human colon carcinoma HCT-116 and SW613-B3 cell lines [22]. C-1 acetoxymethyl—a derivative of 7-deoxypancratistatin—and JC-TH-acetate-4 (JCTH-4) are novel compounds capable of selectively inducing apoptosis and autophagy in human colorectal cancer (CRC) cells alone and in combination with tamoxifen (TAM) [23]. Honokiol (HNK), a biphenolic natural compound, significantly inhibits melanoma cell proliferation, viability, clonogenicity, and induces autophagy on melanoma cells [24]. Isobavachalcone (IBC), a natural chalcone compound, induces apoptosis- and autophagy-related cell death in myeloma cells [25]. In numerous breast cancer studies, MCF-7 is frequently used as a cell model for testing the efficacy of anti-cancer agents. Several natural compounds have been shown to be autophagy inducers, such as rottlerin [26], chrysin-organotin based on chrysin [27], betanin/isobetanin from beetroots [28], cucurbitacin B from cucurbitaceous plants [29], and 2-ethyl-3-*O*-sulphamoyl-estra-1,3,5(10)16-tetraene, a 17-β-estradiol analogue [30]. Additionally, the functions of rottlerin in caspase-3-deficient MCF-7 and caspases-3-transfected MCF-7 are significantly different. In caspase-3-deficient MCF-7, rottlerin acts as an autophagy inducer, while rottlerin acts as an apoptotic activator in caspases-3-transfected MCF-7 [26]. Furthermore, chrysin-organotin [27] and cucurbitacin B [29]. Recently, pancratistatin (PST) analogue, a C-1 acetoxymethyl derivative of 7-deoxypancratistatin (JCTH-4) induced apoptosis and autophagy, and accelerates cell death with combinatorial treatment using time-lapse microscopy in human breast adenocarcinoma and neuroblastoma cells with tamoxifen [31]. Table 1 summarizes our collection of compound names, herb sources, and cancer types to address natural autophagic inducers for anti-cancer activity.

## 3. Underlying Mechanisms of Natural Compound-Induced Autophagy

Accordingly, the mechanisms of natural product actions including plant-derived anti-cancer drug-induced apoptotic cell death may be intrinsic or extrinsic; p53-dependent or -independent pathways; and caspase-dependent or -independent manner in cancer research. Alternative modes of non-apoptotic cell death by plant-derived anti-cancer drugs are emerging, and mainly comprise autophagy, necrosis-like programmed cell death, mitotic catastrophe, and senescence leading to cell death. Considering that non-apoptotic cell death mechanisms of plant-derived anticancer drugs are less reviewed than the apoptotic ones, this article attempts to focus on such alternative cell death pathways for some representative anti-cancer plant-based compounds in clinical development. The understanding of autophagy-induced mechanisms of natural products could afford deep insight into the possibility of exploiting novel molecular pathways and targets of plant-derived compounds for future cancer therapeutics [45]. Various targets or molecules, including sequestosome-1 (SQSTM1), also known as p62, mammalian target of rapamycin (mTOR), uncoordinated-51-like kinase 1 (ULK1), and autophagy-related protein 12 (Atg12), are commonly applied in observations of autophagy, and the Akt/mTOR/p70 ribosomal protein S6 kinase (p70S6K) and the extracellular signal-regulated kinases 1/2 (ERK1/2) pathways are two major pathways that regulate autophagy induced by nutrient starvation [11,46]. In autophagy, rapamycin specifically targets mTOR and further inhibits the activity of mTOR. The inhibition of mTOR subsequently activates Beclin-1 and down-regulates PI3K-ClassIII to finally induce autophagy [47]. In addition to mTOR inhibition, sirolimus (rapamycin) also regulates microRNA expression, which is involved in tumor growth and drug resistance, including miR-7a, miR-17-92, miR-99a, and miR-100 [48]. Some reports have focused on the inhibitory effect of specific cancer cell types and an understanding of mechanical actions in addition to the abovementioned signal pathways, while several other potential signal pathways have been explored (Table 2). On the other hand, we also highlighted three exemplified natural compounds such as curcumin, 16-hydroxycleroda-3,13-dien-15,16-olide, and prodigiosin as interesting autophagy inducers to discuss more facets in the following section.

### 3.1. Curcumin

Curcumin is a yellow pigment isolated from turmeric (*Curcuma longa* L.). Recent study has shown that curcumin provides numerous biological activities including anti-oxidation, antiprotozoal, antimicrobial, immunomodulation, anti-angiogenesis, and antitumor, among others [89]. On anti-tumor activity, curcumin regulates metastasis-relating protein MMP-9 for reducing tumor metastasis [90]. Moreover, curcumin induces G_2_/M phase arresting in glioma cell via upregulation of p21 and ING4, and further induces apoptosis through up-regulating BAX and down-regulating the Bcl-2 and NF-κB signaling pathway in glioma cells [91]. In autophagy, curcumin induces autophagy via the ERK1/2 signaling pathway (Table 2). In glioblastoma, curcumin induces autophagy in vitro and in vivo, and is less toxic to normal cells, especially in glioma-initiating cells (GICs) [92]. A curcumin derivative—2*E*,6*E*-2-(1H-indol-3-yl)methylene)-6-(4-hydroxy-3-methoxybenzylidene)-cyclohexanone (IHCH)—inhibited A549 cell growth and induced the formation of characteristic autophago-lysosomes in a dose- and time-dependent manner [93].

### 3.2. 16-Hydroxycleroda-3,13-dien-15,16-olide (HCD)

*Polyalthia longifolia* var. pendula Linn is popularly known as ulta Ashok in India and is widely grown in gardens in tropical and subtropical Asia, such as the southern part of Taiwan, Pakistan, and Sri Lanka, as an evergreen ornamental tree. Many parts of *P. longifolia* var. pendula Linn tree are important in traditional Indian medicine [94], and encompass various biological functions, such as anti-inflammatory activity in neutrophils, cytotoxicity towards breast cancer cells, and hepatoma cancer cells [95]. The bark has shown to have medicinal value in the treatment of skin diseases, fever, hypertension, diabetes, and helminthiasis [96]. Recently, the chemical components of *P. longifolia* var. pendula such as diterpenes (clerodane and triterpenes) and aporphine alkaloids have been isolated. Diterpenoids in the hexane extract of *P. longifolia* seeds has exhibited significant anti-bacterial and anti-fungal activity [97]. Clerodane diterpenes can induce apoptosis in human leukemia HL-60 cells [98]. Moreover, 16-Hydroxycleroda-3,13-dien-15,16-olide (HCD) and its analogs extracted from the bark of *P. longifolia* have strong anti-inflammatory activity [99]. The enhanced expression of cyto-protective HO-1 factor and anti-inflammatory enzyme in microglia has been reported [100]. Hereby, the induction of apoptosis in leukemia K562 cells via a reduction in histone-modifying enzymes, PRC2-mediated gene silencing, the reactivation of downstream tumor suppressor gene expressions [101], via the PI3K/Akt pathway, and Aurora B resulting in gene silencing and cell cycle disturbance [102]. Our previous studies have demonstrated that HCD could cause apoptosis of two brain cancer cell lines, N18 and C6, via inhibition of focal adhesion kinase (FAK)-related signaling pathway and accordingly induced the autophagic cell death through ROS generation and p38/ERK1/2 signaling pathway activation [103,104]. In oral squamous cell carcinoma, HCD induced autophagy by activating AMPKα and inhibiting Akt, PI3K-ClassIII, and Beclin-1 activity [105].

### 3.3. Prodigiosin (PG)

Prodigiosin (PG, PubChem CID: 5377753) is an alkaloid and natural red pigment, which is a secondary metabolite of *Serratia marcescens* and also from actinomycete bacteria [106]. It is characterized by a common pyrrolyl pyrromethene skeleton [107,108]. The biological role of these pigments in the producer organisms remains unclear. Bacterial PGs and their synthetic derivatives have antimicrobial (bactericidal and bacteriostatic) [109,110,111,112], antimalarial [109,110,113], and antitumor [109,110,114,115,116] activities. In addition, they have been shown to be effective apoptotic agents against various cancer cell lines [117], with multiple cellular targets including multi-drug resistant cells with little or no toxicity towards normal cell lines and induce apoptosis in T and B lymphocytes [118,119]. Recently, PG can induce apoptosis in various cancer cells with low toxicity on normal cells and PG-induced apoptosis may ascribe to Bcl-2 and survivin inhibition in colorectal cancer HT-29 cells [120]. Moreover, PG and its structural analogue (compound R) have induced the expression of p53 target genes accompanied by cell-cycle arrest and apoptosis in p53-deficient cancer cells [121]. A previous study has indicated that PG could be effective as a potential inhibitor compound against COX-2 protein, and can be applied as an anti-inflammatory drug [122]. In melanoma cells, PG activates the mitochondrial apoptotic pathway by disrupting an anti-apoptotic member of the BCL-2 family-MCL-1/BAK complexes by binding to the BH3 domain [123]. Additionally, PG exerts nearly identical cytotoxic effects on the resistant cells in comparison to their parental lines to reveal that this pro-apoptotic agent acts independently on the overexpression of multi-drug resistance transporters—MDR1, BCRP, or MRP [124]. Mechanistically, PG engages the IRE1-JNK and PERK-eIF2α branches of the unfolded protein response (UPR) signaling to up-regulate CHOP that, in turn, mediates BCL2 suppression to induce cell death in multiple human breast carcinoma cell lines [125].

## 4. Perspectives: Natural Autophagy Inducers Potentiate a New Era of Chemotherapeutic Drug Discovery

This review is valuable in terms of clarifying important directions for research on the major role of autophagy inducers resulting in cell death and the underlying mechanisms as seen with numerous natural products. Notably, natural products are an important resource in the discovery of lead compound for anti-cancer drug development, and study on the role of autophagy in the tumor suppressive effects of natural products continues to produce insights into and emerging from difficulties. It is noteworthy that technical variations in detecting autophagy might affect data quality, and study should focus on elaborating the role of natural inducers in deciding cell fate. In vivo study monitoring of natural autophagy inducers in cancer treatment is expected to be a critical effort for the future. Furthermore, the clinically relevant action of autophagy-inducing natural products should be emphasized in translational study.

## Figures and Tables

**Table 1:** Natural autophagic inducers from compound names and herb sources evaluated for various types of cancer.

Compounds	Sources	Cancer Type	Reference
Rdipusilloside I	*Ardisia pusilla* A. DC	Glioblastoma multiforme	[32]
Gossypol	Cotton	Glioblastoma multiforme	[33]
		Breast cancer	[21]
Monanchocidin A	*Monanchora pulchra*	Genitourinary malignancies	[34]
Zerumbone	*Zingiber zerumbet* Smith	Prostate cancer	[35]
Ery5	Magnolol	Prostate cancer	[36]
Cotylenin A + phenethyl isothiocyanate	*Garcinia yunnanensis* Hu	Pancreatic cancer	[37]
Oblongifolin C		Cholangiocarcinoma	[38]
Spicatoside A	*Liriope platyphylla*	Colorectal cancer	[39]
Compound 4h	*Colchicum autumnale*	Colorectal cancer	[40]
Dimethyl cardamonin	*Syzygium samarangense* (Blume) Merr. & L.M. Perry (Myrtaceae)	Colorectal cancer	[41]
JCTH-4	pancratistatin	Colorectal cancer	[23]
Isocryptotanshinone	*Salvia miltiorrhiza*	Lung cancer	[42]
Honokiol	*Magnolia officinalis*	Melanoma	[24]
Isobavachalcone	*Psoralea corylifolia*	Myeloma	[25]
Celastrol	*Tripterygium wilfordii*	Cervical cancer	[43]
Goniothalamin	*Goniothalamus macrophyllus* (Blume) Hook. f. & Thomson var. *macrophyllus*	Renal cancer	[44]

**Table 2:** Natural autophagy inducers and their effect on signal transduction pathways.

Pathways	Compounds	Cancer Type	Reference
Akt	(+)-Grandifloracin	Pancreatic Carcinoma	[49]
PI3K/Akt	Magnolol	Gastric Adenocarcinoma	[50]
	Apigenin	Leukemia	[51]
PI3K/Akt/HK2	Neoalbaconol	Nasopharynx cancer	[52]
Akt/mTOR	Salvianolic Acid B	Colorectal cancer	[53]
	Guttiferone K	Uterus Carcinoma	[54]
	Resveratrol	Melanoma	[55]
	Honokiol	Melanoma	[56]
Akt/mTOR/p70S6K	Curcumin	Glioma	[57]
TR3/Akt2	1-(3,4,5-Trihydroxyphenyl) nonan-1-one	Melanoma	[58]
AMPK	(2R)-kazinol B + Bafilomycin A1	Hepatocarcinoma	[59]
	Oridonin	Colorectal cancer	[60]
AMPK/mTOR	Kazinol A	Bladder cancer	[61]
	Cryptotanshinone	Hepatocarcinoma	[62]
	Tanshinone IIA	Leukemia	[63]
CaMKK/AMPK/mTOR	Alisol B	Nasopharynx cancer	
		Prostate cancer	
Beclin 1	Bromelain + N-acetylcysteine	Gastric Adenocarcinoma	[64,65]
	Paratocarpin E	Breast cancer	[66]
	Evodiamine	Gastric Adenocarcinoma	[67]
	Alma extract	Ovarian cancer	[19]
ERK1/2	Curcumin	Uterine cancer Leiomyosarcoma	[68]
	Curcumin	Glioma	[57]
	Sarsaparilla extract	Gastric Adenocarcinoma	[69]
		Breast cancer	
		Colorectal cancer	
p38/ERK1/2	Resveratrol	Glioma	[70]
p38/JNK	Compound 1 from *Adenophora triphylla* var. *japonica*	Gastric Adenocarcinoma	[71]
Raf/ERK/p90RSK	Tanshinone IIA	Leukemia	[63]
miR-25/ULK1	Isoliquiritigenin	Breast cancer	[72]
NF-κB	Helenalin	Ovarian cancer	[73]
p53	Capsaicin	Lung cancer	[74]
p62	Zosteropenillines 7	Prostate cancer	[75]
p62/LC3-II	Oridonin + NVP-BEZ235	Neuroblastoma	[76]
LC3-II/Atg5/Beclin-1	Resveratrol	Glioblastoma multiforme	[77]
LC3/Atg7/Atg12	Salvigenin	Neuroblastoma	[78]
p-eIF2α	Trehalose	Neuroblastoma	[79]
PERK/eIF2α	Benzyl isothiocyanate	Lung cancer	[80]
ROS	Gelomulide K	Breast cancer	[81]
ROS/JNK	Juglanin	Breast cancer	[82]
	Ginsenoside K	Colorectal cancer	[83]
	Ursolic acid	Colorectal cancer	[84]
ROS/MEK/ERK	CYT-Rx20	Breast cancer	[85]
Wnt/β-catenin	Resveratrol	Breast cancer	[86]
HSF1/Hsp70/ubiquintin	Oridonin	Leukemia	[87]
RelB/p52	Baicalin	Hepatocarcinoma	[88]

AMP-activated protein kinase (AMPK); Calcium/calmodulin-dependent protein kinase kinase (CaMKK); c-Jun N-terminal kinases (JNK); Eukaryotic translation initiation factor 2 subunit 1 (eIF2α); Extracellular signal–regulated kinases (ERK); Heat shock factor 1 (HSF1); Heat shock protein 70 (Hsp70); Hexokinase 2 (HK2); Mammalian target of rapamycin (mTOR); Microtubule-associated protein 1 light chain 3 α (LC3); Mitogen-activated protein kinase kinase (MEK); Nerve growth factor IB (TR3); Nuclear factor κ-light-chain-enhancer of activated B cells (NF-κB); p70 ribosomal protein S6 kinase β-1 (p70S6K); Phosphoinositide 3-kinase (PI3K); Protein kinase B (Akt, PKB); Protein kinase RNA-like endoplasmic reticulum kinase (PERK); Reactive oxygen species (ROS); Sequestosome-1 (SQSTM1); Uncoordinated-51-like kinase (ULK); Wingless/Integrated (Wnt).

## Abbreviations

3-MA	3-Methyladenine
5-FU	5-Fluorouracil
HCD	16-Hydroxycleroda-3,13-dien-15,16-olide
AMPK	AMP-activated protein kinase
CaMKK	Calcium/calmodulin-dependent protein kinase kinase
JNK	c-Jun N-terminal kinases
eIF2α	Eukaryotic translation initiation factor 2 subunit 1
EGCG	Epigallocatechin gallate
ERK	Extracellular signal–regulated kinases
GICs	Glioma-initiating cells
HSF1	Heat shock factor 1
Hsp70	Heat shock protein 70
HK2	Hexokinase 2
HNK	Honokiol
CRC	Human colorectal cancer
IBC	Isobavachalcone
mTOR	Mammalian target of rapamycin
LC3	Microtubule-associated protein 1 light chain 3 α
MEK	Mitogen-activated protein kinase kinase
MGMT	O (6)-methylguanine-DNA methyltransferase
TR3	Nerve growth factor IB
NF-κB	Nuclear factor κ-light-chain-enhancer of activated B cells
OC	Oblongifolin C
p70S6K	p70 ribosomal protein S6 kinase β-1
PST	Pancratistatin
PI3K	Phosphoinositide 3-kinase
PG	Prodigiosin
PCD	Programed cell death
Akt	Protein kinase B
PERK	Protein kinase RNA-like endoplasmic reticulum kinase
Rhiz	Rhizochalinin
ROS	Reactive oxygen species
SQSTM1	Sequestosome-1
TAM	Tamoxifen
ULK	Uncoordinated-51-like kinase
Wnt	Wingless/Integrated
